# JcTI-I: a novel trypsin inhibitor from *Jatropha curcas* seed cake with potential for bacterial infection treatment

**DOI:** 10.3389/fmicb.2014.00005

**Published:** 2014-01-30

**Authors:** Helen P. S. Costa, Jose T. A. Oliveira, Daniele O. B. Sousa, Janne K. S. Morais, Frederico B. Moreno, Ana Cristina O. Monteiro-Moreira, Ricardo A. Viegas, Ilka M. Vasconcelos

**Affiliations:** ^1^Laboratory of Plant Toxins, Department of Biochemistry and Molecular Biology, Federal University of CearaFortaleza, Brazil; ^2^School of Pharmacy, University of FortalezaFortaleza, Brazil; ^3^Department of Forestry Engineering, Federal University of Campina GrandePatos, Brazil

**Keywords:** *Jatropha curcas*, seed cake, serine proteinase inhibitor, trypsin inhibitor, bacterial infections, antimicrobial agent

## Abstract

*Jatropha curcas* seed cake is a low-value by-product resulting from biodiesel production. The seed cake is highly toxic, but it has great potential for biotechnology applications as it is a repository of biomolecules that could be important in agriculture, medicine, and industry. To explore this potential, a novel trypsin inhibitor called JcTI-I was purified by fractionation of the crude extract with trichloroacetic acid (2.5%, v/v) followed by affinity chromatography (Trypsin-Sepharose 4B) and molecular exclusion (Sephacryl S-200). Non-reducing sodium dodecyl sulfate-polyacrylamide gel electrophoresis and gel filtration showed that JcTI-I has approximately 20.0~kDa. Mass spectrometry analysis revealed that the intact molecular mass of JcTI-I is 10.252~kDa. Moreover, JcTI-I is a glycoprotein with 6.4% (m/m) carbohydrates, p*I* of 6.6, N-terminal sequence similarity around 60% to plant albumins and high stability to heat, pH, and salinity. JcTI-I presented antibacterial activity against the human pathogenic bacteria *Salmonella enterica* subspecies *enterica* serovar choleraesuis and *Staphylococcus aureus*, with minimum inhibitory concentration less than 5~μg/mL. Furthermore, JcTI-I did have inhibitory activity against the serine proteases from the tested bacteria. Otherwise, no hemolytic activity of human erythrocytes and signs of acute toxicity to mice were observed for JcTI-I. The results demonstrate the benefits of *J. curcas* seed cake as a source of trypsin inhibitor with potential for biotechnological application as a new antimicrobial agent against human pathogenic bacteria.

## INTRODUCTION

Hospital-acquired infection is the major cause of death in critically ill patients, both in developing and developed countries (Ramsamy et al., 2013). These infections are a challenge to patient safety because the causal agents have developed antibiotic-resistance, thus limiting treatment options (Mulvey and Simor, 2009; Becker et al., 2012). For example, both the methicillin-resistant *Staphylococcus aureus* (MRSA) and multidrug-resistant *Salmonella enterica* are the principal multidrug resistant bacterial pathogens that cause serious community and hospital-acquired infections, responsible for high annual health care costs and psychological stress associated with social stigma (Cosgrove et al., 2003; Brydon et al., 2009; Broughton et al., 2010). Therefore effective new therapeutic agents with novel mechanisms of action for treatment of infections caused by multidrug resistant bacteria are urgently needed (Hughes et al., 2012). In this context, as plants have numerous therapeutic compounds they constitute natural targets from which new antibacterial drugs with high efficacy and less toxicity can be developed to treat infectious diseases (Ngo et al., 2013). Indeed, biologically active compounds from plant resources have been extracted from different species (Savoia, 2012).

*Jatropha curcas*, also known as physic nut, is a shrub belonging to the Euphorbiaceae family. This species is highly adaptable to adverse conditions and is resistant to many pests and pathogens (Debnath and Bisen, 2008; Sabandar et al., 2013). In addition, *J. curcas* seeds constitute an oil-rich plant source from which biodiesel is produced (Rashid et al., 2010). After oil extraction by screw press, the remaining seed cake is highly toxic to a number of animal species, probably due to the presence of phorbol esters and curcin, a type-I ribosome inactivating protein (Goel et al., 2007; Zhao et al., 2012). Protease inhibitor, lectin, and phytate are also present in high amounts (Saetae and Suntornsuk, 2011). Nevertheless, these compounds could be isolated and characterized to exploit their possible medicinal applications, as it was suggested that the *J. curcas* seed cake could be utilized as a source of antibacterial and antifungal agents (Sundari and Selvaraj, 2011).

Among these various molecules present in *J. curcas* seed cake, the protease inhibitors could be a potentially novel class of antimicrobial agents, as they specifically inhibit the catalytic action of enzymes by formation of stoichiometric complex with the target enzymes, blocking or altering its active site (Kim et al., 2009; Volpicella et al., 2011). In fact, protease inhibitors are found to be involved in various important physiological functions like regulators of endogenous proteinases and defense mechanism (Bhattacharjee et al., 2012). However, protease inhibitors also have received new interest due to their biological properties with potential for use as clinical agents. Of importance in the context of seeking plant protease inhibitors as novel therapeutic agents is that Xb-KTI, a Kunitz trypsin inhibitor present in *Xanthosoma blandum* corms with bactericidal activity (Lima et al., 2011). Similarly, the fistulin, a naturally occurring inhibitor of serine protease present in *Cassia fistula* leaves, showed to be very active against several pathogenic bacterial strains, namely, *Staphylococcus aureus*, *Escherichia coli*, *Bacillus subtilis*, and *Klebsiella pneumoniae*, and its efficacy was comparable to the standard drug, streptomycin sulfate (Arulpandi and Sangeetha, 2012).

To the best of our knowledge, a trypsin inhibitor from *J. curcas* seed cake has never been isolated neither its antibacterial activity tested yet. Thus, the present paper describes the purification and physicochemical characterization of a novel trypsin inhibitor from *J. curcas* seed cake, designated JcTI-I. Additionally, to gain better insights on the biological activity of this protein and to devise future use as a new therapeutic drugs, its inhibitory activity against the growth of the human pathogen bacteria *Staphylococcus aureus* and *Salmonella enterica*, as well as the ability of JcTI-I to inhibit the bacterial proteases were evaluated.

## MATERIALS AND METHODS

### MATERIALS

*Jatropha curcas* seed cake was obtained from Instituto Fazenda Tamanduá (Paraíba, Brazil), grounded in a coffee grinder and passed through a 1-mm-mesh screen. The resulting flour was treated with *n*-hexane (1:5, m/v) to remove the remaining oil left after biodiesel extraction. Defatted flour was stored in air-tight containers at 4°C until analysis. The bacteria *Salmonella enterica* subspecies *enterica* serovar choleraesuis (ATCC 10708), *Bacillus subtilis* subspecies *spizizenii* (ATCC 6633), *Pseudomonas aeruginosa* (ATCC 25619), and *Staphylococcus aureus* (ATCC 25923) were obtained from the Department of Biology (UFC), Fortaleza, Brazil. Swiss mice (*Mus musculus*), 20–25 g, were from the animal house at UFC. Azocasein, bovine pancreatic trypsin, bovine pancreatic chymotrypsin, bovine serum albumin (BSA), soybean trypsin inhibitor (SBTI), *N*α-benzoyl-D,L-arginina-*p*-naftilamida (BANA), 4-(dimethylamino)cinnamaldehyde (DMACA), ethylenediaminetetraacetic acid (EDTA), phenylmethylsulfonyl fluoride (PMSF), L-cysteine and *N*α-benzoyl-D,L-arginine 4-nitroanilide hydrochloride (BAPNA), sodium dodecyl sulfate (SDS), molar mass markers, acrylamide, bis-acrylamide, dithiothreitol (DTT) were purchased from Sigma-Aldrich (St. Louis, MO, USA). Chromatographic matrixes were from GE Healthcare. All other chemicals and reagents used were of analytical grade.

### PROTEIN DETERMINATION

The method described by Bradford (1976) was used with BSA as standard. Absorbance at 280 nm was also used to detect the presence of protein in the chromatographic eluates.

### TRYPSIN INHIBITION ASSAY

Trypsin inhibitory activity against bovine trypsin (EC 3.4.21.4) was performed as described by Erlanger et al. (1961) using the chromogenic substrate BAPNA. The enzyme concentration used was established based on a dependent concentration assay and was that producing an absorbance between 0.25 and 0.30 in the enzymatic assay and reaction rate corresponding to ½*V*_max_. The inhibitory assay mixture consisted of 100 μL of crude extract (4.74 mg/mL) or the purified inhibitor (0.03 mg/mL) dissolved in distilled water, incubated with 10 μL of trypsin (0.3 mg/mL in 10^-^^3^ M HCl) and 690 μL of 0.05 M Tris–HCl/0.02 M CaCl_2,_ pH 7.5, at 37°C for 10 min. Next, 500 μL of 1.25 × 10^-^^3^ M BAPNA dissolved in the above buffer were added and the mixture further incubated for 15 min, 37°C. Reaction was stopped by addition of 120 μL of 30% (v/v) acetic acid. The enzymatic activities in the absence and presence of the inhibitor were evaluated by *p*-nitroanilide release from BAPNA measured at 410 nm using a Biochrom Libra S-12 spectrophotometer. One trypsin inhibitory activity unit (TIU) was defined as the decrease in 0.01 U of absorbance per 15 min assay, at 37°C. Appropriate blanks for the enzyme, inhibitor and the substrate were also included in the assay along with the test samples.

### PREPARATION OF THE PROTEIN EXTRACT

Defatted flour was extracted with 0.1 M borate buffer, pH 10.0, in a proportion of 1.0 g of meal to 10.0 mL of buffer for 2 h under sonication, at 4°C. Next the suspension was maintained under constant stirring for 2 h at 4°C and filtered through cheesecloth. The filtrate was centrifuged at 10,000 × *g*, 4°C, 30 min and the clear supernatant, denoted crude extract, was dialyzed (cut-off 12 kDa) against 0.050 M sodium phosphate buffer, pH 7.5. The protein content and trypsin inhibitory activity of this dialyzed extract were determined and it was further used for purification of the trypsin inhibitor as described below.

### PURIFICATION OF *J. curcas* TRYPSIN INHIBITOR

The crude extract prepared as described in Section “protein Determination” was fractionated by precipitation with 2.5% (v/v) trichloroacetic acid (TCA) final concentration, at 4°C and centrifuged at 14,000 × *g*, 4°C, 30 min. The clear supernatant obtained was dialyzed exhaustively against water (Milli-Q grade), lyophilized, and assayed for antitrypsin activity. The TCA fraction (30 mg) was dissolved in 0.050 M sodium phosphate buffer/0.2 M NaCl, pH 7.5 and applied to a trypsin-Sepharose 4B column (11.5 cm × 2.2 cm) equilibrated with the above buffer. After complete removal of the non-retained proteins with the equilibrating buffer, the proteins bound to the immobilized trypsin were eluted with 0.1 M HCl, dialyzed exhaustively against water (Milli-Q grade) and lyophilized. This material (2 mg) was loaded on a Sephacryl S-200 column connected to an ÄKTA-Prime System (GE Healthcare) previously equilibrated and eluted with 0.050 M sodium phosphate buffer/0.2 M NaCl, pH 7.5. Fractions (1 mL) were eluted at the flow rate of 0.5 mL/min and the protein fractions obtained evaluated for trypsin inhibitory activity as described before. The purified trypsin inhibitor was named JcTI-I (*J. curcas* Trypsin Inhibitor I).

### CHARACTERIZATION OF JcTI-I

#### Molecular mass determination

The apparent molecular mass of JcTI-I was determined by denaturing electrophoresis [SDS-polyacrylamide gel electrophoresis (SDS-PAGE); Laemmli, 1970], in 12.5% (m/v) polyacrylamide gels (10 cm × 8 cm). Samples were prepared in 0.5 M Tris–HCl buffer, pH 6.8, containing 1% SDS, in the presence or absence of 1% β-mercaptoethanol and boiled at 98°C, for 5 min, before electrophoresis, which was performed at 20 mA. Protein bands were stained with Coomassie Brilliant Blue G-250 (Candiano et al., 2004). The native molecular mass of JcTI-I (3 mg) was determined by gel filtration on Sephacryl S-200 column, coupled to an ÄKTA-Prime System (GE Healthcare) and equilibrated with 0.050 M sodium phosphate buffer/0.2 M NaCl, pH 7.5. Chromatography was carried out at a constant flow rate of 0.5 mL/min and 1 mL fractions were collected. Before loading on the column, the sample was centrifuged at 14,000 × *g*, 4°C, for 5 min and the supernatant filtered through a Pro-X TM filter unit (0.22 mm hydrophilic cellulose acetate membrane). The column was previously calibrated with proteins of known molecular mass (BSA, 66 kDa; egg albumin, 45 kDa; chymotrypsinogen, 25 kDa; ribonuclease, 13.7 kDa, and aprotinin, 6.5 kDa).

In addition, native mass was obtained by mass spectrometry analysis of JcTI-I (0.1 mg/mL) dissolved in water/acetonitrile (1:1, v/v). The intact mass spectra was acquired in a Synapt G1 HDMS Acquity UPLC instrument (Waters Co., Milford, MA, USA) programmed with a RF *offset* (MS profile) adjusted such that the LC/MS data were effectively acquired from *m*/*z* 400 to 3,000, which allowed to obtain multiply charged mass ions. Analysis was performed using a nanoelectrospray ionization in positive ion mode (ESI+) and a NanoLockSpray source. For all measurements, the mass spectrometer was operated in the “V” mode with a resolving power of at least 10,000 *m*/*z*. The data collection was performed using MassLynx 4.1 software (Waters Co., Milford, MA, USA) and charge distribution spectra were then deconvoluted by the Maximum Entropy Technique (Max-Ent). To assess whether JcTI-I is composed of subunits covalently linked by disulphide bridges it was previously treated with 0.1 M DTT followed by 0.3 M iodoacetamide and trypsin digestion to be analyzed by capillary liquid chromatography/nanoelectrospray ionization tandem mass spectrometry (ESI–LC–MS/MS), using the same equipment as described above, coupled with a nano-high-performance liquid chromatography (UPLC) unit (Waters Co., Milford, MA, USA). The reduced and alkylated JcTI-I was injected using the nanoAcquity UPLC sample manager and the chromatographic separation was performed using an UPLC C18 column (75 μm × 10 cm) with a flow of 0.35 μL/min. The mass spectra were acquired using a data-dependent acquisition (DDA) methodology, where the three top peaks were subjected to MS/MS. Mobile phases A and B consisted of 0.1% formic acid in water and 0.1% formic acid in acetonitrile, respectively. Column was equilibrated with 3% solution A and the gradient conditions used were as follows: 3–40% B for 20 min, 40–70% B for 40 min, 70% for further 50 min and 70-3% of B for 10 min. The data were processed using the Protein Lynx Global Server (Waters Co., Milford, MA, USA) software and the searches made with the assumption that there was a maximum of one missed trypsin cleavage, peptides were mono-isotopic, methionine residues were partially oxidized and cysteine completely carbamidomethylated.

#### Carbohydrate content

The neutral sugar content of JcTI-I-I was estimated by the method of Dubois et al. (1956) using galactose as standard. To 250 μL of JcTI-I-I (0.20 mg/mL), 250 μL of phenol 5% (v/v) and 1.25 mL of concentrated sulfuric acid were added. The mixture was stirred and left at 25°C for 30 min. Readings were taken at 490 nm. The carbohydrate content was estimated based on a calibration curve built with different concentrations of galactose. The value is expressed in percentage (%) of carbohydrate per protein mass. To confirm the presence of covalently bound carbohydrate in JcTI-I a specific staining of the protein bands after SDS-PAGE was carried out (Zacharius et al., 1969). Briefly, after the electrophoresis run the gel was fixed in a solution of 7.5% (v/v) acetic acid for 2 h, followed by incubation in a solution of 0.2% (v/v) periodic acid, at 4°C for 45 min and incubation with the Schiff reagent at 4°C for further 45 min. The glycoprotein nature of the band was revealed by immersion of the gel in a solution of 0.5% (m/v) potassium metabisulfite in 0.05 M HCl.

#### N-terminal sequence analysis

This was established in a Shimadzu PPSQ-10 Automated Protein Sequencer performing Edman degradation. Phenylthiohydantoin (PTH) amino acids were detected at 269 nm after separation on a reversed phase C_18_ column (4.6 mm × 2.5 mm) under isocratic conditions, according to the manufacturer’s instructions. Percentage sequence identity with trypsin inhibitors was searched for automatic alignment, performed using the NCBI-BLAST search system (Altschul et al., 1990).

#### Effect of temperature, pH, and salt

This was evaluated according to Klomklao et al. (2011). JcTI-I (0.035 mg/mL) was diluted with distilled water to obtain 60–70% trypsin inhibition. For thermal stability assay, JcTI-I aliquots were incubated at 90°C for 0, 10, 20, 30, 40, 50, 60, 90, and 120 min and then cooled in ice-water. The trypsin inhibitory activity was determined and reported as the percentage of the residual activity compared to 70% trypsin inhibition. The effect of pH on JcTI-I stability was evaluated by measuring the residual activity after incubation at various pH values for 30 min at 25°C. The buffers used were: 0.05 M glycine-HCl, pH 2.2; 0.05 M sodium acetate, pH 5.2; and 0.1 M sodium borate, pH 10.0. The stability of JcTI-I to salinity was tested by incubating the trypsin inhibitor in NaCl solutions ranging from 0% up to 3% (m/v) concentration, at 25°C for 30 min. After treatment the inhibitory activity against trypsin was determined as previously described and the residual inhibitory activity reported.

#### Enzyme specificity

The papain inhibitory assay was performed as described by Abe et al. (1992), using BANA as substrate. The enzyme concentration used was established based on a dependent concentration assay and was that producing an absorbance between 0.25 and 0.30 in the enzymatic assay and reaction rate corresponding to ½*V*_max_. To 60 μL of papain (0.02 mg/mL in 0.25 M sodium phosphate buffer, pH 6.0), 40 μL of the activation solution (2 × 10^-^^3^ M EDTA + 3 × 10^-^^3^ M DTT, pH 6.0), 200 μL of JcTI-I, and 200 μL of 0.25 M sodium phosphate buffer, pH 6.0, were added. The mixture was incubated for 10 min at 37°C. The reaction was initiated by addition of 200 μL 0.001 × 10^-^^3^ M BANA, prepared in 1% (v/v) dimethyl sulfoxide (DMSO) and 0.025 M sodium phosphate buffer, pH 6.0. After 20 min at 37°C, the reaction was stopped by addition of 500 μL 2% (v/v) HCl in 95% (v/v) ethanol. The color product was developed by adding 500 μL of 0.06% (m/v) DMACA in ethanol and the absorbance was measured at 540 nm. The chymotrypsin inhibitory activity was measured by the method of Erlanger et al. (1961), using azocasein as substrate. Twenty microliters of chymotrypsin (0.1 mg/mL in 2.5 × 10^-^^3^ M HCl) was pre-incubated with JcTI-I (0.2 mg/mL) and 380 μL of 0.05 M Tris–HCl pH 7.5, for 15 min, 37°C. Next, 1% (m/v) azocasein was added to the mixture and incubated for 30 min. Reaction was stopped by addition of 300 μL 20% (v/v) TCA. After centrifugation (10,000 × *g*, 10 min, 25°C), aliquots were withdrawn from the supernatants and added to 2 M NaOH, in an appropriate proportion, and the absorbance taken at 440 nm.

#### IC _50_ value and kinetic studies

The amount of JcTI-I needed for 50% inhibition of trypsin activity was determined as previously described in Section “Trypsin Inhibition Assay,” but using the inhibitor in the concentration range of 1.0 × 10^-^^7^ to 2.0 × 10^-^^7^ M. Kinetic studies of protease inhibition by JcTI-I were conducted according to Bijina et al. (2011), with minor modifications, using bovine trypsin (molecular weight 23.3 kDa) and different concentrations of BAPNA (8.0 × 10^-^^5^ to 1.6 × 10^-^^3^ M). To calculate the kinetic parameters, JcTI-I at 5 × 10^-^^7^ and 10 × 10^-^^7^ M concentrations and 8.0 × 10^-^^6^ M trypsin were previously incubated in 0.05 M Tris–HCl/0.02 M CaCl_2,_ pH 7.5, for 10 min, at 37°C, to reach the assay temperature. Then, 100 μL fractions of JcTI-I were added to 10 μL of the trypsin solution and further incubated for 10 min, at 37°C. Next, both the trypsin solution and trypsin + JcTI-I mixture were added separately to the BAPNA solutions and incubated at 37°C, for 15 min. Reaction was stopped by addition of 120 μL of 30% (v/v) acetic acid. The release of *p*-nitroanilide by the action of trypsin on BAPNA was measured at 410 nm as before. The velocity of enzyme reaction (v) was determined from progress curves using different BAPNA concentrations on the base of product concentrations measured at 410 nm at a fixed time of a reaction. To calculate *K*_m_ (Michaelis–Menten constant) and *V*_max_ (maximal rate) of the reaction and to study the pattern of inhibition, data were plotted as a function of BAPNA concentration using the usual non-linear curve fitting of Michaelis–Menten and linear Lineweaver–Burk, respectively. A secondary plot of 1/*K*_m_ versus JcTI-I concentrations was also drawn and the X-intercept used to calculate the dissociation constant (*K*_i_; Cornish-Bowden, 1995).

### ANTIMICROBIAL ACTIVITY

#### In vitro antibacterial activity

The antibacterial activity (Hancock, 2000) of JcTI-I was tested *in vitro* against *Salmonella enterica*, *Staphylococcus aureus*, *Bacillus subtilis*, and *Pseudomonas aeruginosa*. The pathogenic bacteria were grown to mid-logarithm phase in 5 mL of Mueller–Hinton broth medium, at 37°C. JcTI-I, at a 500 μg/mL final concentration, was prepared in 0.25 M sodium phosphate buffer, pH 7.5, and sterilized using a 0.22 μm membrane, was incubated in 96-well flat microplates (Nunc) with 100 μL of each bacterial suspension (10^5^–10^6^ CFU/mL) dissolved in the growth broth for 4 h at 37°C. Bacterial growth was monitored at 630 nm, every hour within the incubation period, using an automated microplate reader (Bio-TekElx800). The cell growth of both bacteria in the absence of JcTI-I was monitored as a blank control. Experiments were run in triplicate. To determine the minimum inhibitory concentration (MIC), JcTI-I, at 5–500 μg/mL concentration, was dissolved in 0.25 M sodium phosphate buffer, pH 7.5 and 100 μL incubated with equal volume of the bacterial cell suspension (10^5^–10^6^ CFU) in wells of a 96-well polypropylene plate. The plates were kept for 4 h at 37°C and absorbance readings recorded at 630 nm every hour.

#### Activity of JcTI-I against the *Salmonella enterica* and *Staphylococcus aureus* proteases

The capacity of JcTI-I to inhibit the secreted and endogenous bacterial proteases was done following the methodology described by Lima et al. (2011) with minor modifications. *Salmonella enterica* and *Staphylococcus aureus* were cultured in 5 mL of Mueller-Hinton broth overnight, at 37°C, and subcultured in 300 mL of Mueller-Hinton broth, at 37°C and 240 rpm, until the log phase was reached, as measured at 630 nm (Bio-TekElx800 reader). The bacterial cell cultures were collected at the late exponential growth phase by centrifugation at 4,000 × *g*, for 15 min, at 4°C and the pellet resuspended in the protease extraction solution [HCl 0.1% (v/v) + 5.0 × 10^-^^3^ M Tris + 5.0 × 10^-^^3^ M CaCl_2_ + 0.1% (v/v) Triton X-100, pH 7.5] and incubated in ice bath for 15 min. Freezing and thawing took place three times, followed by centrifugation at 4,000 × *g*, for 30 min, at 4°C. The supernatants obtained, containing the protease from *Salmonella enterica* and *Staphylococcus aureus*, were pooled separately for every bacterium species and the serine protease activity as well as the inhibitory activity of JcTI-I assayed, according to the method previously described, using azocasein as substrate, bovine trypsin and PMSF as serine protease control inhibitor.

### HEMOLYTIC ACTIVITY ASSAY

The hemolytic assay, performed in triplicate, was carried out as previously described (Kim et al., 2006), with minor modifications. Human red blood cells were collected in the presence of heparin, washed three times with phosphate-buffered saline (PBS; 3.5 × 10^-^^2^ M phosphate buffer containing 0.15 M NaCl, pH 7.0) and recovered by centrifugation in the same buffer. The hemolytic activity of JcTI-I at 5–500 μg/mL concentration was evaluated by measuring the release of hemoglobin from fresh human erythrocytes. Aliquots (25 μL) of a 2.5% suspension of red blood cells were transferred to 96-well plates and incubated with 25 μL of JcTI-I for 30 min, at 37°C followed by centrifugation at 5,000 × *g*, for 5 min. Hemolysis (%) was determined by measuring the supernatant absorbance at 540 nm. PBS (0.05 M, pH 7.4) and 0.1% (v/v) Triton X-100 were used, respectively, as negative (0% hemolysis) and positive (100% hemolysis) controls.

### TOXICITY ASSAY

The toxicity assay was reviewed and approved by the Animal Ethics Committee (CEPA) of UFC, Brazil, and realized according to the methodology described by Vasconcelos et al. (1994). Toxic activity was defined as mortality observed in Swiss mice within 24 h after intraperitoneal injections of JcTI-I at varied concentrations.

### STATISTICAL ANALYSIS

The results are expressed as the mean ± SEM. The statistical analysis was performed by one-way analysis of variance (ANOVA) followed by Tukey’s multiple comparison tests. The results were considered to be significant at *P* < 0.05.

## RESULTS

### PURIFICATION OF JcTI-I

**FIGURE 1:**
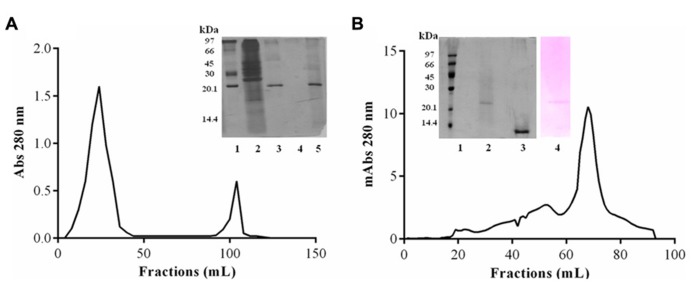
**JcTI-I purification.** **(A)** Affinity-chromatography (Trypsin-Sepharose 4B column). Inset: SDS-PAGE: (1) molar mass markers; (2) crude extract; (3) 2.5% TCA fraction; (4) unbound fractions; (5) bound fractions. **(B)** Size exclusion chromatography (Sephacryl S-200 column) of JcTI-I purified after chromatography on Trypsin-Sepharose 4B column. Inset: SDS-PAGE: (1) molar mass markers; (2 and 3) JcTI-I (5 μg) in the absence and presence of 1% β-mercaptoethanol, respectively; (4) JcTI-I (5 μg) stained with Schiff’s reagent.

Purification of JcTI-I encompassed TCA precipitation of the *J. curcas* protein extract followed by two additional chromatography steps. TCA, at 2.5% (v/v) final concentration, precipitated the proteins with a recovery of up to 35.6% and 29.0-fold purification of the trypsin inhibitor compared to the protein extract (**Table 1**). This TCA precipitated fraction was chromatographed on a trypsin-Sepharose 4B affinity column and the adsorbed proteins, eluted with 0.1 M HCl, concentrated the inhibitory activity that was specific for trypsin (**Figure 1A**), indicating that this chromatography step was important to exclude other protease inhibitors also present in the TCA precipitated fraction, as for papain (data not shown). At this stage, the trypsin inhibitor was purified to 331.5-fold with a protein yield of 1.8% and specific activity of 6.63 × 10^-^^3^ TIU/mg protein (**Table 1**). The fraction obtained from the trypsin-Sepharose 4B affinity chromatography displaying trypsin inhibitory activity was composed of multiple protein bands as evaluated by SDS-PAGE (**Figure 1A**; insert: lane 3). Therefore, this fraction was further purified on a Sephacryl S-200 column (**Figure 1B**). The eluted protein present in the major peak showed a specific activity of 28.35 × 10^-^^3^ TIU/mg protein, 1417.5-fold purification, but a low protein yield of 1.2%. Nevertheless, it was homogeneous by SDS-PAGE as it presented a unique protein band with a relative molecular mass around of 20.0 kDa (**Figure 1B**, insert: lane 2). This purified protein is a trypsin inhibitor of *J. curcas* seed cake that was denominated JcTI-I thereafter.

**Table 1:** Purification steps of a trypsin inhibitor from *J. curcas* cake.

Steps	Total protein^a^(mg)	Total activity^b^(TIU × 10^-3^)	Specificity activity (TIU/mg protein × 10^-3^)	Yield^c^(%)	Purification index^d^
Crude extract	165.6	4.1	0.02	100	1.0
F_2.5_ (TCA)	59.0	34.4	0.58	35.6	29.0
Trypsin-Sepharose 4B	3.0	19.9	6.63	1.8	331.5
Sephacryl S-200	2.0	56.7	28.35	1.2	1417.5

*Results are presented as mean values of three replications.*

a The total amount of protein recovered from 1 g of defatted flour from *J. curcas* seed cake.

b One trypsin inhibitory activity unit (TIU) was defined as the decrease in 0.01 U of absorbance per 15 min assay, at 37^°^C.

c The recovery of protein at each purification step (crude extract, 100%).

d Purification index is calculated as the ratio between the specificity activity obtained at each purification step and that of the crude extract taken as 1.0.

### CHARACTERIZATION OF JcTI-I

#### Molecular mass determination, p*I*, carbohydrate content, and N-terminal sequence

**FIGURE 2:**
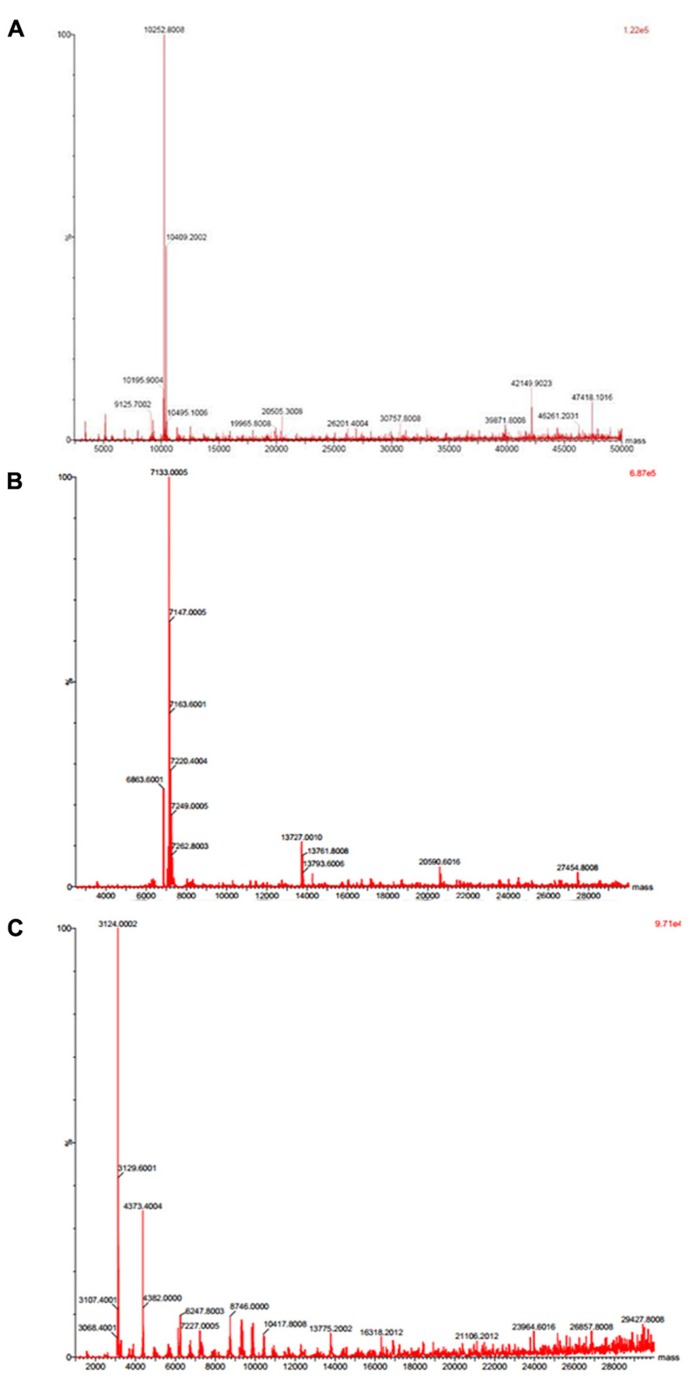
**Mass spectrometry analysis of JcTI-I.** **(A)** Native JcTI-I; **(B,C)** JcTI-I previously reduced and alkylated with DTT and iodoacetamide, respectively.

Native molecular mass of JcTI-I was assessed by gel filtration chromatography on Sephacryl S-200 column. A molecular mass of 20.2 kDa was calculated for the inhibitor dissolved in 0.05 M sodium phosphate buffer, pH 7.5, containing 0.2 M NaCl. This value is very similar compared to the molecular mass obtained by SDS-PAGE in the absence of reducing agents (**Figure 1B**, insert: lane 2). However, ESI mass spectrometry under native conditions, with JcTI-I dissolved in water/acetonitrile (1:1, v/v), revealed a 10.252 kDa polypeptide (**Figure 2A**) which is very close to the molecular mass of JcTI-I found under denaturing condition in the presence of β-mercaptoethanol (**Figure 1B**; insert: lane 3) and after two-dimensional (2D) gel electrophoresis that also showed a p*I* of 6.6 (data not shown) for the purified inhibitor. DTT reduced and alkylated JcTI-I produced two derived peptides of 7.133 and 3.124 kDa, after MS/MS analysis, suggesting that these polypeptides are linked by disulfide bond (**Figures 2B,C**, respectively). Moreover, JcTI-I has 6.4% covalently linked carbohydrate (**Figure 1B**; insert: lane 4) and an N-terminal sequence of VRDICKKEAERRDLSSCENYITQRRGY (**Table 2**). This sequence was confirmed after mass spectrometry analysis of JcTI-I (**Table 3**). Alignment of this N-terminal sequence with known sequences in the NCBI non-redundant database showed 68 and 58% similarity with an allergenic 2S albumin from *J. curcas* seeds and with an albumin 2S from *Ricinus communis*, respectively. In addition, other peptide sequences were obtained similar to 2S albumins, particularly from *R. communis* (**Table 3**). However, no similarity was found between the N-terminal sequence of JcTI-I generated by Edman degradation and the peptide sequences obtained by MS/MS analysis with known members of the protease inhibitor family.

**Table 2:** Comparison of N-terminal sequence of JcTI-I with similar protein sequences.

Protein	Species	N-terminal sequence	Identity (%)	Access number ^a^
JcTI-I	*Jatropha curcas*	VRDICKKEAERRDLSSCENYITQRRGY		C0HJF7^b^
Jat c 1^c^	*Jatropha curcas*	VRDKCGEEAERRTLXGCENYISQRR	68	
Precursor albumin 2S	*Ricinus communis*	CRQEIQRKDLSSCEQYIRQSSSRR	58	XP_002522851
Glycine-rich protein	*Arabidopsis thaliana*	DIAKKKTKKKDLSSSDNYFTKR	55	NP_683354
Short chain of napin-like peptide	*Ricinus communis*	CRQEVQRKDLSSCERYLRQSSSRR	54	AAB50869
Short chain of napin-like peptide	*Momordica charantia*	REQLRSCESFLRQSRGY	53	AAB50872

a Results of a BLAST search.

b UniProt accession number of JcTI-I.

c Deduced amino acid sequence of Jat c 1^b^, a allergenic 2S albumin (Maciel et al., 2009).

**Table 3:** Peptide sequences of JcTI-I identified after data-dependent acquisition (DDA) analysis.

*m/z*	Charge	Delta (ppm)	Sequence	Modifications
821.3901	2	4.091931	(R)RDLSSCENYITQR(R)	Carbamidomethyl C (6)
743.3438	2	10.359877	(R)DLSSCENYITQR(R)	Carbamidomethyl C (5)
424.7177	2	-2.8809798	(K)ELSAICR(C)	Carbamidomethyl C (6)
646.3268	2	7.850316	(R)CESIHYLLEK(Q)	Carbamidomethyl C (1)
774.3472	2	2.6834285	(K)QLEEGEVGSEDEAR(R)	

#### Thermal and pH stability

**FIGURE 3:**
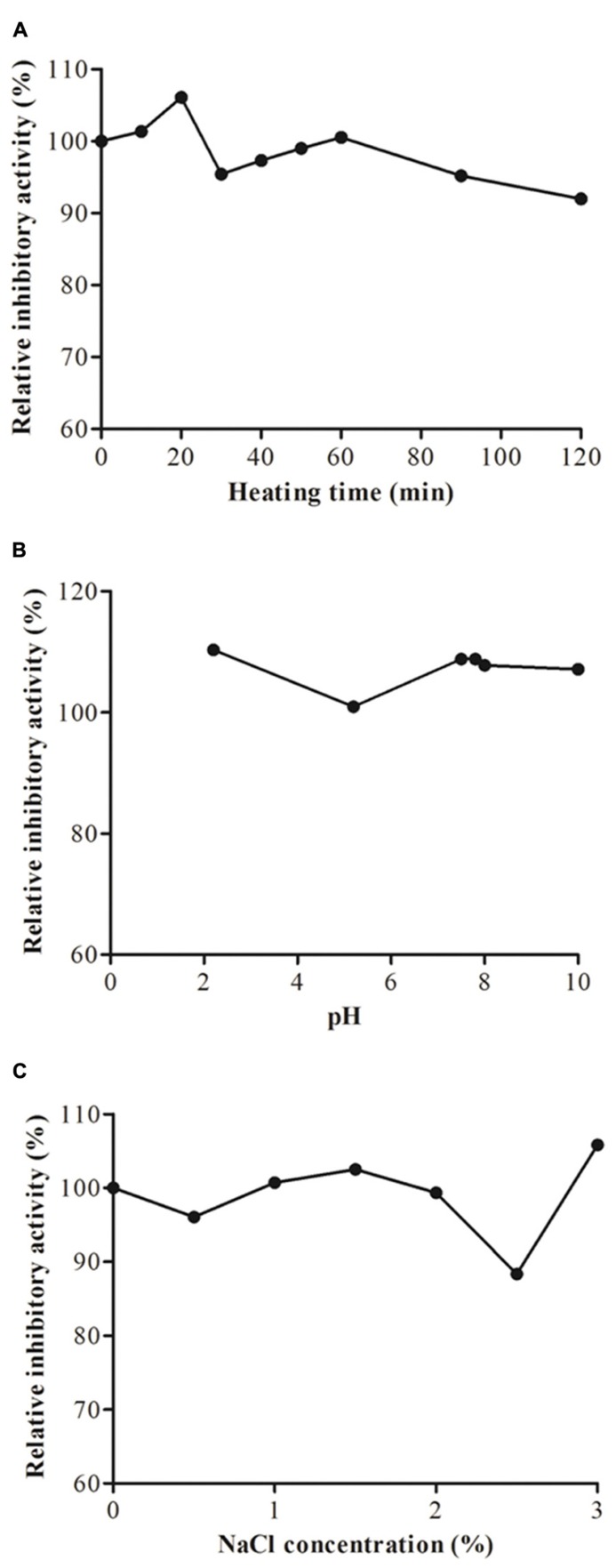
**Trypsin inhibitory activity assay of JcTI-I at pH 7.5, at room temperature (around 25°C) after: **(A)** heat-treatment; **(B)** incubation at pH 2–10 for 30 min; **(C)** incubation with 0–3 M NaCl concentration.** Each point represents the mean of three estimates. The standard deviation was less than 10%.

Heating of JcTI-I at 90°C for up to 20 min induced a slight increased in the inhibitory activity upon trypsin as compared to 70% trypsin inhibition of the native inhibitor (**Figure 3A**). Afterward the heat treatment for up to 120 min had little consequence on the inhibitory effect of JcTI-I as it was reduced only to around 4–5% of that of the untreated inhibitor. Incubation of JcTI-I at the pH range of 2.0–10.0 (**Figure 3B**) had overall a slight inducing effect (10%) on inhibition (**Figure 3B**), except at pH 5.0 that maintained 70% trypsin inhibition. Incubation of JcTI-I with different concentrations of salt (0–3%) showed that the protein retained approximately its original activity up to 2.0% NaCl concentration (**Figure 3C**). However, at 2.5% NaCl concentration an 11.6% reduction in the inhibitory activity was noticed (**Figure 3C**), whereas at 3.0% the trypsin inhibition was 10% higher than the 70% inhibition induced by the untreated inhibitor.

#### Enzyme specificity

JcTI-I was specific for inhibiting bovine trypsin, but did not inhibit significantly chymotrypsin and papain.

#### IC _50_ value and kinetic parameters

**FIGURE 4:**
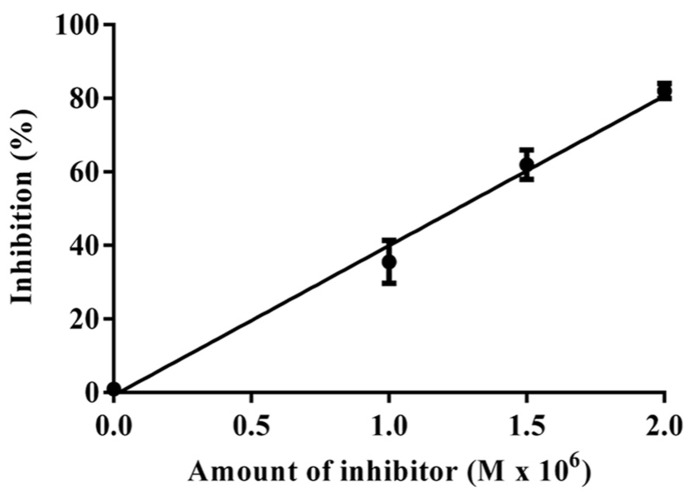
**Stoichiometry of the JcTI-I-protease interaction.** Bar indicates standard deviation from triplicate determinations.

**FIGURE 5:**
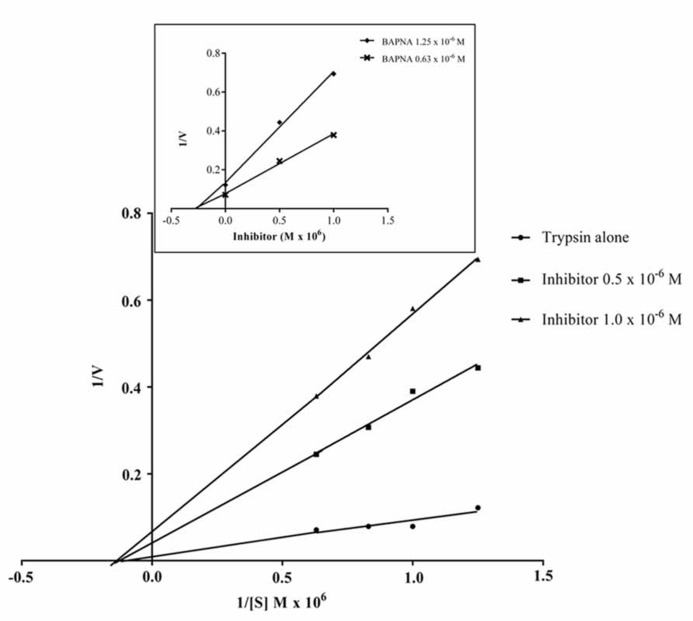
**Lineweaver–Burk plot (1/*v* × 1/[S]) of the inhibition of trypsin by JcTI-I, at two different inhibitor concentrations, in conjunction with the Dixon plot (1/*v* × [I], insert) for identification of *K*_**m**_, *K*_**i**_ and the likely mechanism of enzyme inhibition.** Each point represents the mean of three estimates. The standard deviation was less than 10%.

The data obtained for the studies conducted on protease–protease inhibitor interaction is depicted in **Figure 4**. The amount of inhibitor needed for 50% trypsin inhibition (IC_50_) was 1.25 × 10^-^^6^ M. Moreover, it is calculated that the stoichiometry of trypsin–protease inhibitor interaction is 1:1.7. Kinetic studies of inhibition of trypsin by JcTI-I showed that plots of a fixed concentration of trypsin (8.0 × 10^-^^6^ M) preincubated with the buffer alone (control) and with 0.5 and 1.0 × 10^-^^6^ M of JcTI-I produced different slopes of 1/*v* versus 1/[s] (**Figure 5**). These data indicated a non-competitive mechanism of action of JcTI-I as lines are converging to the same point on the 1/[S] axis where *K*_m_ is unchanged, whereas *V*_max_ is reduced. Furthermore, calculated *K*_i_ (**Figure 5**, insert) was on average 0.24 × 10^-^^6^ M, indicating that inhibition of the trypsin hydrolysis by JcTI-I occurred at very low concentration characterizing a very potent inhibitor.

### ANTIMICROBIAL ACTIVITY OF JcTI-I

**FIGURE 6:**
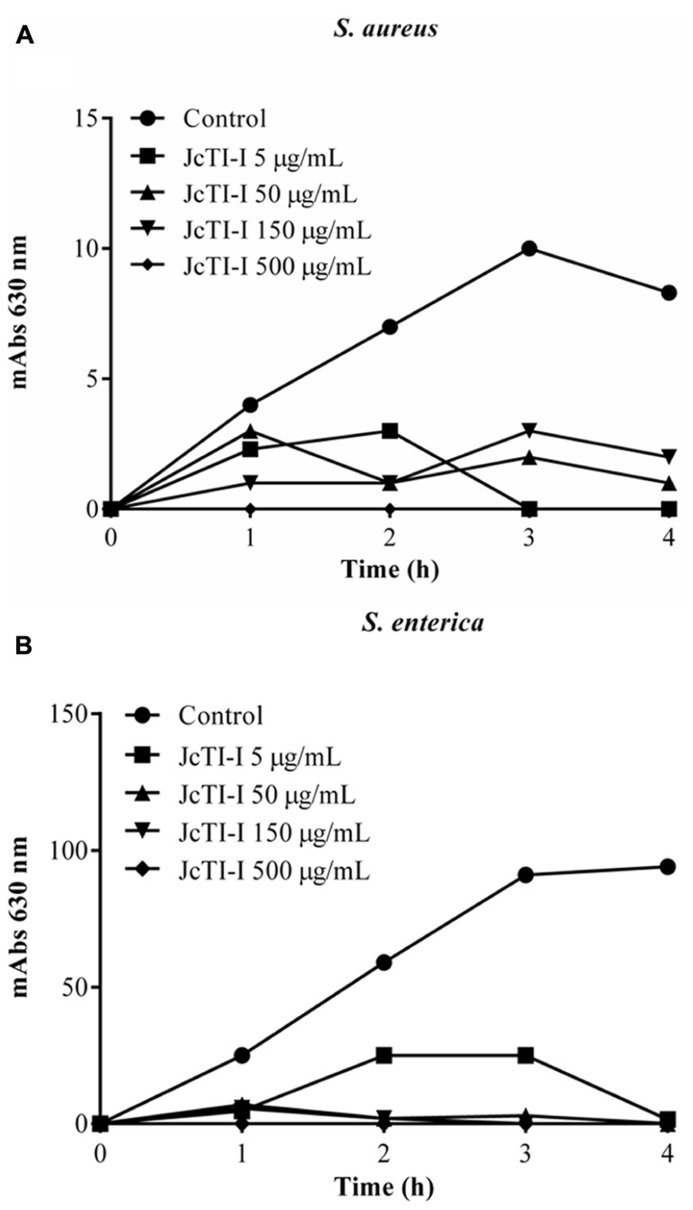
**Effect of JcTI-I at different concentrations on the growth of *Staphylococcus aureus* (A), and *Salmonella enterica* (B).** Control: 0.05 M sodium phosphate buffer (pH 7.5). The minimal inhibitory concentration (MIC) of JcTI-I was determined based on these data. Assay was done in triplicate for every inhibitor concentration. The standard deviation was less than 10%.

**FIGURE 7:**
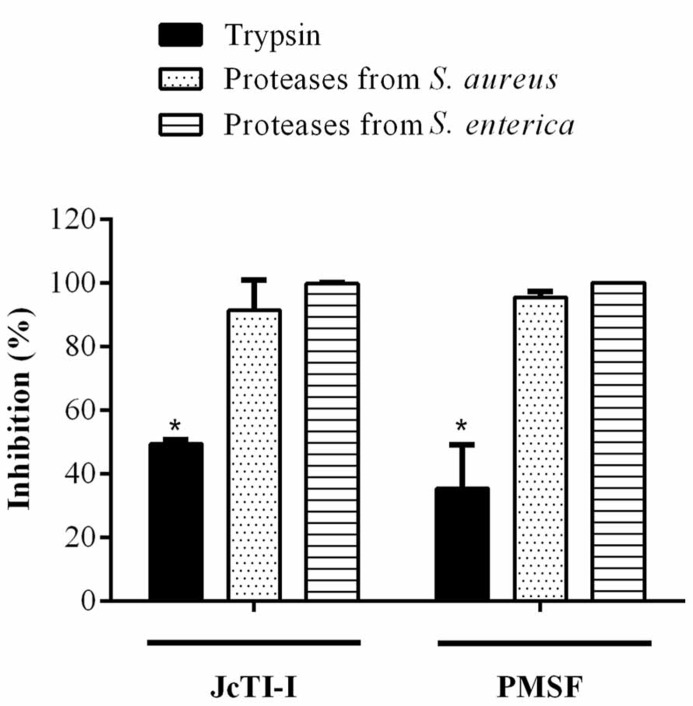
**Inhibitory effect (%) of JcTI-I on the proteases extracted from *Staphylococcus aureus* and* Salmonella enterica*.** Asterisks represent significant difference (*P* < 0.05) in comparison to control (0.25 M sodium phosphate buffer, pH 7.5). The experiments were run in triplicate and each value is presented as mean ± standard deviation.

JcTI-I did not affect the growth of *Bacillus subtilis* and *Pseudomonas aeruginosa*, even at a concentration of 500 μg/mL (data not shown). However, the protein inhibited the growth of *Staphylococcus aureus* and *Salmonella enterica* at all concentrations tested when compared to controls. The MIC was calculated as 5 μg/mL for both bacteria (**Figures 6A,B**). Moreover, JcTI-I caused 84.6% and almost 100% inhibition of the proteases extracted from *Staphylococcus aureus* and *Salmonella enterica*, respectively, values similar to that found for protein sparing modified fast (PSMF; **Figure 7**).

### ASSESSMENT OF HEMOLYTIC ACTIVITY OF JcTI-I

**FIGURE 8:**
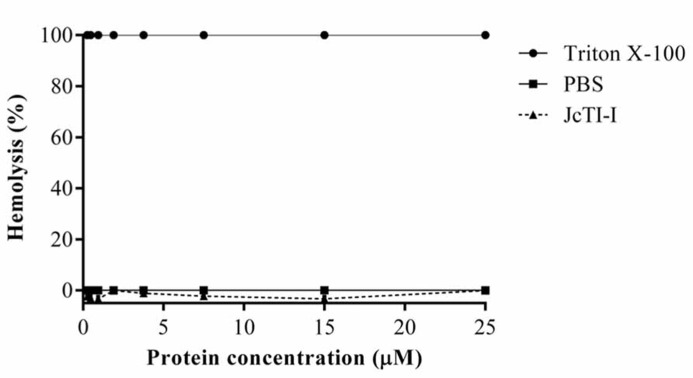
**Assessment of the hemolytic effect of JcTI-I on human erythrocytes.** A suspension of washed human erythrocytes was incubated with various concentrations of JcTI-I (-▲-). Values (OD_540_) were normalized against 100% lysed erythrocytes (0.1% Triton X-100) (-•-). Control was the red cells incubated with 0.05 M PBS, pH 7.4 (-▪-). Each point represents the mean of three estimates. The standard deviation was less than 10%.

JcTI-I did not promote hemolysis of human erythrocyte under the experimental conditions tested (**Figure 8**).

### ASSESSMENT OF TOXIC ACTIVITY OF JcTI-I

JcTI-I did not display toxic activity to mice, even at a concentration 100 times greater than the MIC for *Staphylococcus aureus* and *Salmonella enterica*.

## DISCUSSION

Purification of a novel trypsin inhibitor (JcTI-I) from *J. curcas* seeds cake was achieved by TCA precipitation of the protein extract followed by two chromatographic steps. JcTI-I is a 10.252 kDa neutral glycosylated protein with a p*I* of 6.6 and 6.4% of carbohydrate. Both the molecular mass and p*I* are similar to other seed trypsin inhibitors previously characterized as, for example, the 10.0 kDa trypsin inhibitor from *Zea mays* (Baker et al., 2009) and that purified from *Phaseolus limensis* seeds, with p*I* of 7.6 (Wang and Rao, 2010).

Analyzing the results of both SDS-PAGE and MS/MS, it appears that the 10.252 kDa JcTI-I molecule is composed of a 7.133 and 3.124 kDa polypeptides jointed together by at least one disulphide bridge and that DTT + iodoacetamide treatments, but not β-mercaptoethanol alone, were able of irreversibly splitting the molecule in the small and large subunits. The results of size exclusion chromatography of JcTI-I on Sephacryl S-200 gave a 20.2 kDa molecule. It is plausible that under such mild conditions in which JcTI-I was dissolved in 0.050 M sodium phosphate buffer containing 0.2 M NaCl, pH 7.5, the 10.252 kDa monomer associated to form a 20.2 kDa dimer, which does not occur in the presence of acetonitrile in the MS/MS analysis.

Alignments of JcTI-I sequence with other protein sequences showed that the highest similarity was verified with Jat c 1, a *J. curcas* allergen that belongs to the 2S albumin family (Maciel et al., 2009), but not with known protease inhibitor sequences. Several trypsin inhibitors show sequence similarities with the 2S albumin-like proteins, as two barley Bowman–Birk type trypsin inhibitor isoforms and the trypsin inhibitor of *Brassica juncea* seeds (Terras et al., 1993; Mandal et al., 2002). Therefore, JcTI-I might be a novel trypsin inhibitor that belongs to the 2S albumin family.

Protease inhibitors exhibit a considerable stability to high temperatures and to large pH variations (Bhattacharyya et al., 2006; Bijina et al., 2011). JcTI-I has also these common properties as it was active and stable after incubation at 90°C for 60 min and in the pH range 2.0–10.0, in agreement with other plant protease inhibitors. Moreover, JcTI-I was stable to high salt concentrations as it retained full activity after treatment with up to 2% NaCl. The trypsin inhibitor purified from adzuki bean (*Vigna angularis*) seeds was also stable when incubated with NaCl up to 3% concentration (Klomklao et al., 2011). Such stability was associated to disulphide bridges that are presumably responsible for the functional stability of Kunitz type protease inhibitors in the presence of physical and chemical denaturants such as temperature, pH, and reducing agents (Kridric et al., 2002).

JcTI-I is a non-competitive trypsin inhibitor similar to other trypsin inhibitors, such as AETI, APTI, and TTI from the seeds of *Archidendron ellipticum* (Bhattacharyya et al., 2006), *Adenanthera pavonina* (Macedo et al., 2004), and *Tamarindus indica* (Araújo et al., 2005), respectively. The IC_50_ of JcTI-I for trypsin was 1.25 × 10^-^^6^ M, indicating that it was more potent than EvTI, the trypsin inhibitor from *Erythrina velutina* seeds (IC_50_ = 2.2 × 10^-^^6^ M; Machado et al., 2013) and less than SSTI2, the trypsin inhibitor from *Sapindus saponaria* seeds (IC_50_ = 8.3 × 10^-^^8^ M; Macedo et al., 2011). JcTI-I interaction with trypsin occurred at the 1:1.7 molar ratio, indicating a 1:2 stoichiometry, with two equivalent binding sites. Similar result was found for a protease inhibitor from *Moringa oleifera* leaves (Bijina et al., 2011). The inhibition constant (*K*_i_) for JcTI-I was 2 × 10^-^^11^ M under the assay conditions. Low *K*_i_ values were also found for the trypsin inhibitors CBTI-2 from *Caesalpinia bonduc* (Bhattacharyya et al., 2007) and AETI (Bhattacharyya et al., 2006) with *K*_i_ of 2.4 and 2.7 × 10^-^^10^ M, respectively.

Resistance of some strains of Gram-positive and Gram-negative bacteria to conventional antibiotics has increased dramatically caused or induced by the widespread misuse and overuse of antibiotics and represents a serious threat to public health worldwide. Therefore, discovery and/or development of alternative, non-conventional drugs with activity against most resistant bacteria for infection control are of paramount importance (Lima et al., 2011). *J. curcas* is a plant traditionally used for medicinal purposes and its antimicrobial potential has been previously reported. Indeed, extracts from leaves, root barks, latex, and various fruit parts of *J. curcas* showed inhibitory activity against *E. coli*, *Staphylococcus aureus*, *Pseudomonas aeruginosa*, and *Bacillus subtilis* (Arekemase et al., 2011; Nyembo et al., 2012; Rachana et al., 2012). However, few reports are related to the antimicrobial activity of compounds present in the seed cake of *J. curcas*, although its potential use as a source of antibacterial agents has been suggested (Sriprang et al., 2010; Sundari and Selvaraj, 2011). The seed cake of *J. curcas* represents an attractive raw material to extract novel antibacterial drugs because it is produced in large scale as a waste material after oil extraction to produce biodiesel. It is estimated that 1-tn per day of *J. curcas* extracted oil generates, approximately, 3 tn of seed cake (Srividhya et al., 2010).

The use of protease inhibitors as novel therapeutics has previously been proposed (Bhattacharjee et al., 2012; Machado et al., 2013). Therefore, take into consideration the huge amount of *J. curcas* seed cake generated, from which JcTI-I was purified, the hypothesis that it could behave as a natural drug against human pathogens was also tested. This was experimentally confirmed as JcTI-I behaved as a potent inhibitor of *Staphylococcus aureus* and* Salmonella enterica* growth. The MIC of JcTI-I for both bacteria was 5 μg/mL. This concentration is sixfold lower compared with that found for a potato inhibitor effective against various bacteria, including *Staphylococcus aureus* with a MIC around 30 μg/mL (Kim et al., 2006), about 50-fold lower than that of Xb-KTI, a kunitz proteinase inhibitor from *X. blandum* corms, active against *Salmonella typhimurium*, with a MIC of about 256 μg/mL (Lima et al., 2011) and about 25-fold lower than that of cloxacillin, a β-lactam antibiotic used as therapeutic drug against MRSA with a MIC around 128 μg/mL (Islam et al., 2008). According to Hancock (2000), the best protein/peptide candidates for antibacterial drugs have MICs ranging from 1 to 8 μg/mL. Based on these results, JcTI-I can be considered as a potent inhibitor of bacterial growth and could be explored as an antibiotic protein to help bring down the 10–30% mortality caused by *Staphylococcus aureus* (van Hal et al., 2012).

Serine proteases have been implicated in the virulence of some bacterial strains (Speranskaya et al., 2006; Tripathi et al., 2011). Therefore, substances that can interfere with the proteolytic activity of these enzymes, like protease inhibitors, could be an effective strategy to combat bacterial infections (González-Lamothe et al., 2009). JcTI-I did have inhibitory activity against the serine proteases from the tested bacteria. It caused about 85 and 100% inhibition of the proteases extracted from *Staphylococcus aureus* and *Salmonella enterica*, respectively, values compared to those achieved by PSMF, a synthetic inhibitor. These results are similar to that reported by Lima et al. (2011) who found that Xb-KTI caused about 80% inhibition of the serine proteases from *Salmonella typhimurium*.

To assess whether JcTI-I promotes lysis of eukaryotic cells, this protease inhibitor was incubated with human erythrocytes up to the concentration of 500 μg/mL, about 100-fold higher than the MIC of JcTI-I (5 μg/mL) for both bacteria. Even at such concentration, JcTI-I does not lysis the red cells. This result suggests that the mode of action of JcTI-I is not by disrupting cell membranes and it does not have toxic effects toward mammalian cells. In addition, JcTI-I was not toxic to mice at a concentration very much higher than the MIC for *Staphylococcus aureus* and *Salmonella enterica*.

In summary, we have isolated, purified, and characterized a novel trypsin inhibitor from *J. curcas* seed cake, named JcTI-I, which possesses a potent activity against the human pathogenic bacteria *Staphylococcus aureus* and *Salmonella enterica*. The lack of hemolytic activity against human erythrocytes and toxic activity to mice together with resistance to heat treatment, pH, high salt concentrations, and putative resistance to proteases make JcTI-I a pharmacologically interesting and valuable drug for the design of a novel antibiotic medicament.

## Conflict of Interest Statement

The authors declare that the research was conducted in the absence of any commercial or financial relationships that could be construed as a potential conflict of interest.

## References

[B1] Abe M. Abe K. Kuroda M. Arai S. (1992). Corn kernel cysteine proteinase inhibitor as a novel cystatin superfamily member of plant origin: molecular cloning and expression studies. *Eur. J. Biochem.* 209 933–937 10.1111/j.1432-1033.1992.tb17365.x 1425699

[B2] Altschul S. F. Gish W. Miller W. Myers E. W. Lipman D. J. (1990). Basic local alignment search tool. *J. Mol. Biol.* 215 403–410 2231712 10.1016/S0022-2836(05)80360-2

[B3] Araújo C. L. Bezerra I. W. L. Oliveira A. S. Moura F. T. Macedo L. L. P. Gomes C. E. M. (2005). In vivo bioinsecticidal activity toward *Ceratitis capitata* (fruit fly) and *Callosobruchus maculatus* (*Tamarindus indica*). *J. Agric. Food Chem.* 53 4381–4387 10.1021/jf0502505 15913299

[B4] Arekemase M. O. Kayode R. M. O. Ajiboye A. E. (2011). Antimicrobial activity and phytochemical analysis of *Jatropha curcas* plant against some selected microorganisms. *Int. J. Biol.* 3 52–59 10.5539/ijb.v3n3p52

[B5] Arulpandi I. Sangeetha R. (2012). Antibacterial activity of fistulin: a protease inhibitor purified from the leaves of *Cassia fistula*. *ISRN Pharm.* 2012 84073 10.5402/2012/584073 PMC3388288 22779011

[B6] Baker R. L. Brown R. L. Chen Z.-Y. Cleveland T. E. Fakhoury A. M. (2009). A maize trypsin inhibitor (ZmTIp) with limited activity against *Aspergillus flavus*. *J. Food Prot.* 72 185–188 19205484 10.4315/0362-028x-72.1.185

[B7] Becker P. Santos O. Castrucci F. M. Dias C D’Azevedo P. A. (2012). First report of methicillin-resistant *Staphylococcus aureus* Cordobes/Chilean clone involved in nosocomial infections in Brazil. *Epidemiol. Infect.* 140 1372–1375 10.1017/S095026881100210X 22008212

[B8] Bhattacharjee C. Prasad D. T. Manjunath N. H. Sanyal D. Zarga S. M. (2012). Exploring plant proteinase inhibitors. *Genomics Appl. Biol.* 3 8–21 10.3969/gab.2012.03.0002

[B9] Bhattacharyya A. Mazumdar S. Leighton S. Babu C. (2006). A Kunitz proteinase inhibitor from *Archidendron ellipticum* seeds: purification, characterization, and kinetic properties. *Phytochemistry* 67 232–241 10.1016/j.phytochem.2005.11.010 16376957

[B10] Bhattacharyya A. Rai S. Babu C. R. (2007). A trypsin and chymotrypsin inhibitor from *Caesalpinia bonduc* seeds: isolation, partial characterization and insecticidal properties. *Plant Physiol. Biochem.* 45 169–177 10.1016/j.plaphy.2007.02.003 17400464

[B11] Bijina B. Chellappana S. Basheera S. M. Elyasa K. K. Bahkalic A. H. Chandrasekarana M. (2011). Protease inhibitor from *Moringa oleifera* leaves: isolation, purification, and characterization. *Process Biochem.* 46 2291–2300 10.1016/j.procbio.2011.09.008

[B12] Bradford H. H. (1976). A rapid and sensitive method for the quantification of microgram quantities of protein using the principle of protein dye binding. *Anal. Biochem.* 72 248–254 10.1016/0003-2697(76)90527-3 942051

[B13] Broughton E. I. Ip M. Coles C. L. Walker D. G. (2010). Higher hospital costs and lengths of stay associated with quinolone-resistant *Salmonella enterica* infections in Hong Kong. *J. Public Health (Oxf.)* 32 165–172 10.1093/pubmed/fdp057 19528063

[B14] Brydon L. Walker C. Wawrzyniak A. J. Chart H. Steptoe A. (2009). Dispositional optimism and stress-induced changes in immunity and negative mood. *Brain Behav. Immun.* 23 810–816 10.1093/pubmed/fdp057 19272441 PMC2715885

[B15] Candiano G. Bruschi M. Musante L. Santucci L. Ghiggeri G. M. Carnemolla B. (2004). Blue silver: a very sensitive colloidal Coomassie G-250 staining for proteome analysis. *Electrophoresis* 25 327–1333 10.1002/elps.200305844 15174055

[B16] Cornish-Bowden A. (1995). *Fundamentals of Enzyme Kinetics*, 3rd Edn. London: Portland Press

[B17] Cosgrove S. E. Sakoulas G. Perencevich E. N. Schwaber M. J. Karchmer A. W. Carmeli Y. (2003). Comparison of mortality associated with methicillin-resistant and methicillin-susceptible *Staphylococcus aureus* bacteremia: a meta-analysis. *Clin. Infect. Dis.* 36 53–59 10.1086/377611 12491202

[B18] Debnath M. Bisen P. S. (2008). *Jatropha curcas* L., a multipurpose stress resistant plant with a potential for ethnomedicine and renewable energy. *Curr. Pharm. Biotechnol.* 9 288–306 10.2174/138920108785161541 18691089

[B19] Dubois M. Gilles K. A. Hamilton J. K. Rebers P. A. Smith F. (1956). Colorimetric method for determination of sugars and related substances. *Anal. Chem.* 28 350–354 10.1021/ac60111a017

[B20] Erlanger B. F. Kolowsky M. Cohen W. (1961). The preparation and properties of two new chromogenic substrates of trypsin. *Arch. Biochem. Biophys.* 95 271–278 10.1016/0003-9861(61)90145-X 13890599

[B21] Goel G. Makkar H. P. Francis G. Becker K. (2007). Phorbol esters: structure, biological activity, and toxicity in animals. *Int. J. Toxicol.* 26 279–288 10.1080/10915810701464641 17661218

[B22] González-Lamothe R. Mitchell G. Gattuso M. Diarra M. S. Malouin F. Bouarab K. (2009). Plant antimicrobial agents and their effects on plant and human pathogens. *Int. J. Mol. Sci.* 10 3400–3419 10.3390/ijms10083400 20111686 PMC2812829

[B23] Hancock R. E. (2000). Cationic antimicrobial peptides: toward clinical applications. *Expert Opin. Investig. Drugs* 9 1723–1729 10.1517/13543784.9.8.1723 11060771

[B24] Hughes D. F. Devocelle M. Humphreys H. (2012). Beyond conventional antibiotics for the future treatment of methicillin-resistant *Staphylococcus aureus* infections: two novel alternative. *FEMS Immunol. Med. Microbiol.* 65 399–412 10.1111/j.1574-695X.2012.00954.x 22409572

[B25] Islam M. A. Alam M. M. Choudhury M. E. Kobayashi N. Ahmed M. U. (2008). Determination of minimum inhibitory concentration (MIC) of cloxacillin for selected isolates of methicillin-resistant *Staphylococcus aureus* (MRSA) with their antibiogram. *Bangladesh J. Vet. Med.* 6 121–126

[B26] Kim J.-Y. Park S.-C. Hwang I. Cheong H. Nah J.-W. Hahm K.-S. (2009). Protease inhibitors from plants with antimicrobial activity. *Int. J. Mol. Sci.* 10 2860–2872 10.3390/ijms10062860 19582234 PMC2705521

[B27] Kim M. H. Park S. C. Kim J. Y. Lee S. Y. Lim H. T. Cheong H. (2006). Purification and characterization of a heat-stable serine protease inhibitor from the tubers of new potato variety ``Golden Valley''. *Biochem. Biophys. Res. Commun.* 346 681–686 10.1016/j.bbrc.2006.05.186 16777063

[B28] Klomklao S. Benjakul S. Kishimura H. Chaijan M. (2011). Extraction, purification and properties of trypsin inhibitor from Thai mung bean (*Vigna radiate* (L.) R. Wilczek). *Food Chem.* 129 1348–1354 10.1016/j.foodchem.2011.05.029

[B29] Kridric M. Fabian H. Brzin J. Popovic T. Pain R. H. (2002). Folding, stability and secondary structure of a new cysteine dimeric proteinase inhibitor. *Biochem. Biophys. Res. Commun.* 297 962–967 10.1016/S0006-291X(02)02328-8 12359248

[B30] Laemmli U. K. (1970). Cleavage of structural proteins during the assembly of the bacteriophage T4. *Nature* 227 680–685 10.1038/227680a0 5432063

[B31] Lima T. B. Silva O. N. Migliolo L. Souza-Filho C. R. Gonçalves E. G. Vasconcelos I. M. (2011). A Kunitz proteinase inhibitor from corms of *Xanthosoma blandum* with bactericidal activity. *J. Nat. Prod.* 74 969–975 10.1021/np200312r 21520894

[B32] Macedo M. L. de Sá C. M. Freire M. D. Parra J. R. (2004). A Kunitz-type inhibitor of coleopteran proteases, isolated from *Adenanthera pavonina* L. seeds and its effect on *Callosobruchus maculatus*. *J. Agric. Food Chem.* 52 2533–2540 10.1021/jf035389z 15113152

[B33] Macedo M. L. Diz Filho E. B. Freire M. G. Oliva M. L. Sumikawa J. T. Toyama M. H. (2011). A trypsin inhibitor from *Sapindus saponaria* L. seeds: purification, characterization, and activity towards pest insect digestive enzyme. *Protein J.* 30 9–19 10.1007/s10930-010-9296-7 21127952

[B34] Machado R. J. Monteiro N. K. Migliolo L. Silva O. N. Pinto M. F. Oliveira A. S. (2013). Characterization and pharmacological properties of a novel multifunctional Kunitz inhibitor from *Erythrina velutina* seeds. *PLoS ONE * 8:e63571 10.1371/journal.pone.0063571 PMC3666885 23737945

[B35] Maciel F. M. Laberty M. A. Oliveira N. D. Felix S. P. Soares A. M. Verïcimo M. A. (2009). A new 2S albumin from *Jatropha curcas* L. seeds and assessment of its allergenic properties. *Peptides* 30 2103–2107 10.1016/j.peptides.2009.08.008. 19686791

[B36] Mandal S. Kundu P. Roy B. Mandal R. K. (2002). Precursor of the inactive 2S seed storage protein from the Indian mustard *Brassica juncea* is a novel trypsin inhibitor. Characterization, post-translational processing studies, and transgenic expression to develop insect-resistant plants.* J. Biol. Chem.* 277 37161–37168 10.1074/jbc.M205280200 12147695

[B37] Mulvey M. R. Simor A. E. (2009). Antimicrobial resistance in hospitals: how concerned should we be? *CMAJ* 180 408–415 10.1503/cmaj.080239 19221354 PMC2638041

[B38] Ngo L. T. Okogun J. I. Folk W. R. (2013). 21st century natural product research and drug development and traditional medicines. *Nat. Prod. Rep.* 30 584–592 10.1039/c3np20120a 23450245 PMC3652390

[B39] Nyembo K. Kikakedimau N. Mutambel H. Mbaya N. Ekalakala T. Bulubulu O. (2012). In vitro antibacterial activity and phytochemical screening of crude extracts from *Jatropha curcas* Linn. *Eur. J. Med. Plants* 2 242–251

[B40] Rachana S. Tarun A. Rinki R. Neha A. Meghna R. (2012). Comparative analysis of antibacterial activity of *Jatropha curcas* fruit parts. *J. Pharm. Biomed. Sci.* 15 1–4

[B41] Ramsamy Y. Muckart D. J. J Han K. S. S. (2013). Microbiological surveillance and antimicrobial stewardship minimise the need for ultrabroad-spectrum combination therapy for treatment of nosocomial infections in a trauma intensive care unit: an audit of an evidence-based empiric antimicrobial policy. *S. Afr. Med. J.* 103 371–376 10.7196/SAMJ.6459. 23725954

[B42] Rashid U. Anwar F. Jamil A. Bhatti H. N. (2010). *Jatropha curcas* seed oil as a viable source for biodiesel. *Pak. J. Bot.* 42 575–582

[B43] Sabandar C. W. Ahmat N. Jaafar F. M. Sahidin I. (2013). Medicinal property, phytochemistry and pharmacology of several *Jatropha* species (Euphorbiaceae): a review. *Phytochemistry* 85 7–29 10.1016/j.phytochem.2012.10.009 23153517

[B44] Saetae D. Suntornsuk W. (2011). Toxic compound, anti-nutritional factors and functional properties of protein isolated from detoxified *Jatropha curcas* seed cake. *Int. J. Mol. Sci.* 12 66–77 10.3390/ijms12010066 21339978 PMC3039944

[B45] Savoia D. (2012). Plant-derived antimicrobial compounds: alternatives to antibiotics. *Future Microbiol.* 7 979–990 10.2217/fmb.12.68 22913356

[B46] Speranskaya A. S. Krinitsina A. A. Revina T. A. Gerasimova N. G. Keruchen’ko Y. S. Shevelev A. B. (2006). Heterologous expression, purification, and properties of a potato protein inhibitor of serine proteinases. *Biochemistry* 71 1176–1182 10.1134/S0006297906110022 17140378

[B47] Sriprang S. Sriprang N. Sumpradit T. Shimbhu D. (2010). Antibacterial activities of crude extracts from physic nut (*Jatropha curcas*) seed residues. *Sci. Asia* 36 346–348 10.2306/scienceasia1513-1874.2010.36.346

[B48] Srividhya K. P. Tamizharasan T. Jayaraj S. Muralledharan C. (2010). Characterization and gasification using *Jatropha curcas* seed cake. *J. Biofuels* 1 30–36 10.5958/j.0976-3015.1.1.005

[B49] Sundari J. Selvaraj R. (2011). Antibacterial and antifungal activity of seed extract from *Jatropha curcas* Linn. *Int. J. Curr. Res.* 3 84–87

[B50] Terras F. R. G. Torrenkens S. Van Leuven F. Osborn R. W. Vanderleyden J. Cammue B. P. A. (1993). A new family of basic cysteine-rich plant antifungal proteins from Brassicaceae species. *FEBS J.* 316 233–240 10.1016/0014-5793(93)81299-f 8422949

[B51] Tripathi V. R. Kumar S. Garg S. K. (2011). A study on trypsin, *Aspergillus flavus* and *Bacillus* sp. protease inhibitory activity in *Cassia tora* (L.) syn *Senna tora* (L.) Roxb. seed extract. *BMC Complement. Altern. Med.* 11:56 10.1186/1472-6882-11-56 PMC3144011 21749682

[B52] van Hal S. J. Jensen S. O. Vaska V. L. Espedido B. A. Paterson D. L. Gosbell I. B. (2012). Predictors of mortality in *Staphylococcus aureus* bacteremia. *Clin. Microbiol. Rev.* 25 362–386 10.1128/CMR.05022-11 22491776 PMC3346297

[B53] Vasconcelos I. M. Trentin A. Guimarães J. A. Carlini C. R. (1994). Purification and physicochemical characterization of soyatoxin, a novel toxic protein isolated from soybeans (*Glycine max*). *Arch. Biochem. Biophys.* 312 357–366 10.1006/abbi.1994.1320 8037448

[B54] Volpicella M. Leoni C. Costanza A. De Leo F. Gallerani R. Ceci L. R. (2011). Cystatins, serpins and other families of protease inhibitors in plants. *Curr. Protein Pept. Sci.* 12 386–398 10.2174/138920311796391098 21418017

[B55] Wang S. Rao P. (2010). A leguminous trypsin-chymotrypsin inhibitor *Limenin* with antifungal activity from *Phaseolus limensis*. *Eur. Food Res. Technol.* 231 331–338 10.1007/s00217-010-1285-8 PMC7079880 32214901

[B56] Zacharius R. M. Zell T. E. Morrison J. H. Woodlock J. J. (1969). Glycoprotein staining following electrophoresis on acrylamide gels. *Anal. Biochem.* 30 148–152 10.1016/0003-2697(69)90383-2 4183001

[B57] Zhao Q. Wang W. Wang Y. Xu Y. Chen F. (2012). The effect of curcin from *Jatropha curcas* on apoptosis of mouse sarcoma-180 cells. *Fitoterapia* 83 849–52 10.1016/j.fitote.2012.03.005 22445551

